# Predictors of vigorous exercise adoption and maintenance over four years in a community sample

**DOI:** 10.1186/1479-5868-1-13

**Published:** 2004-09-01

**Authors:** Kerri N Boutelle, Robert W Jeffery, Simone A French

**Affiliations:** 1 Division of General Pediatrics and Adolescent Health, University of Minnesota, 200 Oak St. SE, Suite 160, Minneapolis, MN 55455, USA; 2 Division of Epidemiology, School of Public Health, University of Minnesota, 1300 S. Second St, Suite 300, Minneapolis, MN 55455, USA

## Abstract

**Background:**

Very little is known about the correlates of adoption and maintenance of vigorous exercise. The purpose of this study was to understand the sociodemographic correlates of exercise adoption and maintenance in a community sample.

**Methods:**

917 women and 229 men completed annual surveys as part of a community-based weight gain prevention trial over four years. Multivariate regressions evaluated predictive factors for maintenance of vigorous exercise over time in regular exercisers, and predictors of adoption of exercise in adults who were sedentary at baseline.

**Results:**

Exercise maintenance at Years 2 and 3 was associated with ethnicity and exercise level at baseline, while exercise maintenance at Year 4 was associated with television watching, BMI and exercise at baseline. Exercise level at baseline was associated with exercise initiation at Year 2 and Year 3. Income level, marital status, and smoking status predicted exercise initiation at Year 4.

**Conclusions:**

Predictors of vigorous exercise maintenance were more consistent than predictors of vigorous exercise initiation. Results suggest that those who adopt vigorous exercise are a heterogeneous group and intervention messages could be more broadly focused. These data also suggest that exercise maintenance interventions should continue to target low-income populations with messages regarding smoking, weight and television. Clearly further research is needed to understand the factors that contribute to exercise initiation and maintenance, and to develop effective interventions to improve levels of physical activity levels.

## Background

Vigorous exercise is considered one of the key-components of a healthy lifestyle and cardiovascular fitness. Higher levels of exercise are associated with lower risks of hypertension, diabetes, osteoporosis, colon cancer, coronary heart disease, depression, anxiety, and has been shown to enhance weight control [1-3]. While earlier public health recommendations targeted vigorous physical activity, current recommendations include both vigorous and moderate physical activity [4]. Current vigorous activity recommendations include "increasing the proportion of adults who engage in vigorous physical activity that promotes the development and maintenance of cardiorespiratory fitness three or more days per week for 20 or more minutes per occasion" [5]. Although the benefits of exercise are well documented, research shows that only 15–25% of adults are engaging in vigorous exercise three or more times a week for at least 20 minutes [2,6]. More than 60% of American adults are not exercising at a moderate or vigorous level frequently enough to reap the health benefits [2]. Nearly 25% of all U.S. adults report no leisure time exercise at all [6]. 

Numerous studies have examined predictors of exercise maintenance among participants in organized exercise programs [7]. However, data using longitudinal community based studies are sparse. Two community studies that examined the maintenance and adoption of exercise showed that maintenance of vigorous activity over one year was associated with attitudes toward exercise, exercise knowledge, female gender, and self-efficacy [8]. Over two years, vigorous physical activity maintenance was associated with self-efficacy and younger age for initially active men and with education for initially active women [9]. Adoption of moderate activity over one year was associated with health knowledge [8]. Adoption of vigorous activity over two years was associated with self-efficacy, younger age, and neighborhood environment in men, and education, self-efficacy, and friend and family support in women [9].

Although more research is being conducted on correlates of adoption and maintenance of exercise, more longitudinal research is needed among demographically diverse community samples [10]. Considering only 10–25% of community residents have been successful in adopting even short-term leisure time exercise [8,11,12] there is a need to learn more about the people in the community who adopt and continue exercising. Research on potential correlates of long-term maintenance, initiation, and change in these correlates is needed to better understand how changes in life circumstances might be associated with changes in vigorous exercise. 

The present study sought to cross-sectionally and prospectively examine the correlates of regular vigorous exercise in a sample of community adults over a four year period. Specifically, this study attempted to expand on the current literature by understanding the relationship between regular vigorous exercise and exercise adoption and maintenance by evaluating three comparisons in a community sample: 1) cross-sectional differences in characteristics of regular vigorous exercisers compared to non-exercisers, 2) predictive factors for maintenance of vigorous exercise over time in people who are already exercising at a high level as compared to those who do not maintain their exercise level, and 3) predictors of adoption of exercise in community adults who were sedentary at baseline as compared to those who do not adopt vigorous exercise. 

## Methods

### Sample

The study sample included 917 women and 229 men who completed baseline surveys as part of a 3-year community-based weight gain prevention trial (Pound of Prevention; POP). Participants were recruited using a variety of methods, including direct mail sent to university employees, and advertisements in community newspapers, health department employee newsletters and radio public service announcements. In addition, recruitment also targeted lower-income women at commercial shopping centers and at community health department clinics. Lower income women were paid $20 to enroll in the study. All participants were informed that they would be randomly assigned to either a mail-based educational program, or a no contact control group and that they would be measured once per year for a total of 4 years. This study was approved by the University of Minnesota Institutional Review Board.

### Measures

Study participants completed questionnaires and were measured for height and weight at baseline and at three annual data collection visits (Years 2, 3 and 4) following baseline. Measures included in the present study are listed below.

#### Vigorous exercise

Planned vigorous exercise behavior was assessed using 5 questions from a self-administered version of the Physical Activity History questionnaire (PAH). The PAH is reliable and valid and has been used in several large epidemiologic studies [13]. For this study, exercise activities were limited to those that would deliver significant cardiovascular benefit. Participants rated how frequently in the past year they had engaged in one or more of the activities listed. Response choices on the PAH included; never or less than once per month; 1–3 per month; 1–2 per week; 3–4 per week; or 5 or more per week. Five questions were used in this study to represent high intensity planned exercise; 1) Vigorous jogging, running, backpacking or mountain climbing, 2) Bicycle faster than 10 MPH or exercise hard on an exercise bicycle or rowing machine, 3) Vigorous exercise class or vigorous dance, 4) Brisk walking, hiking, skating or cross-country skiing or 5) other vigorous exercise (including lap distance swimming, vigorous racket sports and other strenuous sports such as competitive basketball, football, volleyball and soccer).

#### Hours spent watching television

To evaluate competing activities, we evaluated participant's report of time spent watching television. Time spent watching television has been positively correlated with body weight, presumably in part due to its displacement of exercise behaviors [14]. Participants reported the number of hours they watch television on an average day.

#### Social Support

Social support from family and friends has been found to be related to exercise in a number of studies [9]. Participants in this study ranked both their family and friends on the extent to which they were supportive of healthy eating and exercise behaviors on a 5 point scale, with 1 representing "Not at all helpful" to 5 representing "Very helpful".

#### Body Mass

Height was measured to the nearest centimeter using a wall-mounted ruler and weight was measured to the nearest half pound using calibrated balance beam scales. Body mass index was calculated using the formula weight (kg)/height (m2). 

#### Demographic Information

All demographic information was self-reported and included age, educational attainment (highest level completed), gender, employment status, income, ethnicity, smoking status and marital status.

### Statistical Analysis

Before performing analyses evaluating the predictors of exercise maintenance and initiation, the possibility of an intervention effect was evaluated. Maintenance of high intensity exercise at Year 2 was not significantly associated with treatment, however, maintenance of high intensity exercise at Years 3 and 4 were associated with treatment status. Intervention participants were more likely to be maintaining exercise behavior at Year 3 (X^2 ^= 14.14, p = .042) and at Year 4 (X^2 ^= 6.42, p = .011). Initiation of exercise at Year 2 and Year 3 were not significantly associated with treatment status. However, initiation of exercise at Year 4 was marginally positively associated with treatment (X^2 ^= 3.30, p = .069). Due to these associations, treatment assignment (intervention vs control) was controlled in multiple regression analyses.

Participants were classified as regular vigorous exercisers (> 3 times per week) and non-regular exercisers (< 3 times per week) based on their responses to the five planned high-intensity exercises on the PAH at each of the 4 assessment points. Using these classifications (yes/no vigorous exercise), the participants were classified as maintainers if they exercised more than three times per week at both baseline and at a subsequent annual evaluation (Year 2, 3, or 4), and non-maintainers if they exercised three times per week at baseline and less than three times per week at a subsequent annual evaluation (Year 2, 3, or 4). Similarly, participants were classified as initiators if they reported less than three exercise sessions per week at baseline and more than three exercise sessions per week at subsequent annual evaluation (Year 2, 3, or 4), and non-initiators if they exercised less than three times per week at both baseline and a subsequent annual evaluation (Year 2, 3, or 4).

Descriptive analyses using t-tests and chi-square tests were utilized to assess univariate associations between the baseline predictor variables and exercise status. Maintainers were compared with non-maintainers, and consistently sedentary participants were compared with participants who adopted vigorous activity at Years 2, 3 and 4. Logistic regression was used to further assess the statistical significance of associations between the predictor variables and exercise maintenance and adoption. These univariate logistic regression models predicted exercise maintenance (yes/no) and exercise initiation (yes/no) at Years 2, 3 and 4 using the baseline demographic factors as independent variables. 

Furthermore, the variables that were significant in the univariate analyses were entered into a multivariate logistic regression model to assess the relative weight and statistical significance of associations between the predictor variables and the variables representing exercise maintenance and initiation. These multivariate logistic regression models predicted exercise maintenance (yes/no) and exercise initiation (yes/no) at Years 2, 3 and 4. In these multivariate regressions, we controlled for exercise level at baseline and treatment status. All statistical analyses were conducted using the SAS Version 6.12 [15].

Since other research studies have indicated gender differences in predictors of exercise maintenance and initiation [16], the gender by exercise interaction was evaluated in a regression model using the frequency of vigorous exercise per week at Year 2 as the dependent variable. The independent variables in this model were the main effects for gender, the main effect for vigorous exercise at baseline, and the gender by vigorous exercise at baseline interaction. The interaction was not significant and thus the data were not stratified for analyses. 

## Results

### Cross-sectional sample description

Using the definition of vigorous exercise described above, 564 participants (453 females and 111 males) vigorously exercised more than 3 times per week and 582 participants (464 females and 118 males) did not vigorously exercise more than 3 times per week at baseline. Sociodemographic characteristics of these participants are described in Table 1. Not surprisingly, participation in vigorous exercise was associated with being employed, non-smoker, lower BMI, fewer hours spent watching television, and higher perceived social support from family and friends. None of the other demographic variables were significantly associated with vigorous exercise at baseline. 

**Table 1:** Correlates of exercise status at baseline

		**Exercisers < 3 X/wk at baseline N = 582**	**Exercisers > 3 X/wk at baseline N = 564**
**Gender**	Female	464 (79.7%)	453 (80.3%)
**Age (years)**		37.6 (SD = 6.3)	37.6 (SD = 7.2)
**BMI (kg/m2)**		28.1 (SD = 6.3)	26.2 (SD = 5.2)***
**Employed**	Yes	474 (81.4%)	495 (87.8%)
**Ethnicity**	White	500 (85.9%)	503 (89.1%)
**Income group**	< 25 K	218 (37.5%)	190 (33.8%)
**Marital Status**	Married	288 (49.5%)	266 (47.2%)
	Sep/Div/Wid	96 (16.5%)	96 (17.0%)
	Never Married	198 (34.0%)	202 (35.8%)
**Education**	HS Degree or less	73 (12.54%)	70 (12.4%)
	Some college	235 (40.4%)	192 (34.0%)
	College degree or more	274 (47.1%)	302 (53.6%)
**Smoking**	Yes	131 (22.5%)	80 (14.2%)***
**TV hours/day**		2.6 (SD = 2.5%)	2.1 (SD = 1.9%)
**Social support family**		2.7 (SD = 1.3)	2.9 (SD = 1.3)
**Social support friend**		2.6 (SD = 1.2)	3.0 (SD = 1.2)***

*** denotes significant difference at the p < .001

### Prospective analyses evaluating predictors of exercise maintenance

#### Prospective univariate evaluations of maintenance of exercise at the three annual evaluations

Maintainers and non-maintainers were compared on baseline demographic variables, social support, hours spent watching television and smoking. Similar patterns of associations were found for annual visits at Years 2, 3 and 4. Participants who maintained their exercise level at evaluation Years 2, 3 and 4 weighed less, were employed, Caucasian, of a higher income group, more highly educated, more likely to be non-smokers and watched less television per day at baseline. At evaluation Years 3 and 4, the maintainers were older than the non-maintainers at baseline. The maintainers and non-maintainers did not report differences in marital status or social support. These results are presented in Table 2. 

**Table 2:** Associations between exercise maintenance and baseline demographic, smoking, social support and hours watching television.

		**Year 2**		**Year 3**		**Year 4**	
		Maintain N = 378	Non-maintain N = 186	Maintain N = 277	Non-maintain N = 230	Maintain N = 216	Non-maintain N = 256
**Gender**	Female	300 79.4%	153 82.3%	214 77.3%	194*** 84.4%	170 78.7%	209 81.6%
**Age (years)**		38.0 SD = 6.6	36.9 SD = 8.4	38.3 SD = 6.6	36.9** SD = 8.0	38.7 SD = 6.5	37.1** SD = 7.9
**BMI (kg/m2)**		25.5 SD = 4.7	27.8*** SD = 6.0	25.7 SD = 4.7	26.7** SD = 5.8	25.4 SD = 4.6	27.0*** SD = 5.8
**Employed**	Yes	341 90.2%	154*** 82.8%	250 90.3%	197 85.7%	200 92.6%	217*** 84.8%
**Ethnicity**	White	350 92.6%	153*** 82.3%	259 93.5%	192*** 83.5%	203 94.0%	222*** 86.7%
**Income Group**	< $25,000	111 29.4%	79*** 42.5%	82 29.6%	89** 38.9%	57 26.4%	108*** 42.2%
**Marital Status**	Married	181 47.9%	85 45.7%	130 46.9%	108 47.0%	106 49.1%	115 44.9%
	Sep/Div/ Widowed	59 15.6%	37 19.9%	42 15.2%	39 17.0%	35 16.2%	49 19.1%
	Never Married	111 29.4%	79 42.5%	105 37.9%	83 36.1%	75 34.7%	92 35.9%
**Education**	HS or less	35 9.26%	35 18.8%	23 8.3%	40 17.4%	15 6.9%	41 16.0%
	HS + some college	128 33.8%	64 34.4%	93 33.6%	79 34.4%	73 33.8%	96 37.5%
	College or more	215 56.9%	87*** 46.8%	161 58.1%	111 48.3%	128 59.3%	119*** 46.5%
**Smoking**	yes	42 11.1%	38*** 20.4%	29 10.5%	39** 16.7%	24 11.1%	45** 17.6%
**TV/day**		1.8 SD = 1.8	2.5*** SD = 2.20	1.78 SD = 1.6	2.4*** SD = 2.1	1.7 SD = 1.5	2.4*** SD = 2.1
**Social support family**		3.0 SD = 1.4	2.8 SD = 1.2	3.0 SD = 1.4	2.9 SD = 1.3	3.0 SD = 1.4	2.9 SD = 1.3
**Social support friend**		3.0 SD = 1.2	3.1 SD = 1.2	3.0 SD = 1.2	3.0 SD = 1.2	3.0 SD = 1.2	3.0 SD = 1.2

** denotes significant difference at the p < .05

*** denotes significant difference at the p < .001

#### Prospective multivariate predictors of exercise maintenance

Multivariate logistic regression analyses were performed to examine the predictors exercise maintenance at Years 2, 3 and 4. Demographic variables at baseline (BMI, income group, employment status, education, ethnicity, smoking status, hours watched TV/day, age, gender) were entered into logistic regression model along with exercise level at baseline and treatment group. Exercise maintenance at Years 2, 3 and 4 were the dependent variables in the three models. At Year 2, ethnicity (OR = .52, CI = .27–.99), BMI (OR = .93, CI = .89–.96) and exercise at baseline (OR = 1.21, CI = 1.11–1.31) were significant predictors of exercise maintenance. Ethnicity (OR = .463, CI = 0.229–0.933) and exercise at baseline (OR = 1.21, CI = 1.12–1.31) were significant predictors of exercise maintenance at Year 2. Television hours per day (OR = 0.86, CI = .74–.99), BMI (OR = .95, CI = .91–.99) and exercise at baseline (OR = 1.22, CI = 1.13–1.31) were significant predictors of exercise maintenance at Year 4.

### Prospective analyses evaluating predictors of exercise initiation

#### Univariate evaluations of initiation of exercise at the three annual evaluations

Results of univariate analyses relating exercise initiation with baseline variables are shown in Table 3. Participants who initiated a vigorous exercise program differed from consistently sedentary persons in ways that were similar to those observed for exercise maintenance. These results, however, were weaker in magnitude and not as consistent over the three time periods as the results from the exercise maintenance analyses. At baseline, initiators at Year 2 had a higher income, reported higher social support from family and friends, and had a lower BMI. The only significant difference between the initiators and the sedentary persons was hours watching television per day at Year 3. Finally, initiators at Year 4 were more likely to be married at baseline. 

**Table 3:** Associations between exercise initiation and baseline demographic, smoking, social support and hours watching television.

		**Year 2**		**Year 3**		**Year 4**	
		Adopt N = 135	Sedentary N = 447	Adopt N = 73	Sedentary N = 347	Adopt N = 48	Sedentary N = 326
**Gender**	Female	108 80.0%	356 79.6%	51 69.9%	305*** 81.5%	36 75.0%	269 82.5%
**Age (years)**		37.9 SD = 6.3	37.5 SD = 6.3	38.2 SD = 5.2	37.4 SD = 6.5	37.4 SD = .6.7	37.4 SD = 6.6
**BMI (kg/m2)**		27.34 SD = 6.1	28.4* SD = 6.3	27.6 SD = 5.5	28.5 SD = 6.4	28.3 SD = 6.9	28.6 SD = 6.4
**Employed**	Yes	114 84.4%	360 80.5%	63 86.3%	297 79.4%	38 79.5%	259 79.5%
**Ethnicity**	White	118 87.4%	382 85.5%	63 86.3%	319 85.3%	42 87.5%	277 84.5%
**Income Group**	< $25,000	41 30.4%	177** 39.6%	26 35.6%	151 40.4%	20 41.7%	131 40.2%
**Marital Status**	Married	69 51.1%	219 49.0%	33 45.2%	186 49.7%	30 62.5%	156 47.9%
	Sep/Div/ Widowed	22 16.3%	74 16.5%	13 17.8%	61 16.3%	9 18.8%	52 16.0%
	Never Married	44 32.59%	154 34.5%	27 37.0%	127 34.0%	9 18.8%	18.8* 36.2%
**Education**	HS degree or less	14 10.4%	59 13.2%	7 9.6%	52 13.9%	5 10.4%	47 14.4%
	HS degree + some college	49 36.3%	186 41.6%	25 34.3%	161 43.1%	19 39.6%	142 43.6%
	College degree + more	72 53.3%	202 45.2%	41 56.2%	161 43.1%	24 50.0%	137 42.0%
**Smoking status**	yes	25 18.5%	106 23.7%	15 20.6%	91 24.3%	6 12.5%	85** 26.1%
**TV/day**		2.4 SD = 1.9	2.7 SD = 2.6	2.1 SD = 1.6	2.8*** SD = 2.8	2.6 SD = 3.2	2.8 SD = 2.7
**Social support family**		3.0 SD = 1.3	2.7*** SD = 1.3	2.6 SD = 1.4	2.7 SD = 1.3	2.9 SD = 1.2	2.6 SD = 1.3
**Social support friend**		2.8 SD = 1.2	2.6** SD = 1.2	2.5 SD = 1.1	2.6 SD = 1.2	2.5 SD = 1.2	2.6 SD = 1.2

*** denotes significant difference at the p < .001

** denotes significant difference at the p < .05

#### Prospective multivariate predictors of exercise initiation

Logistic regression analyses were also performed to examine the predictors of exercise initiation and Years 2, 3 and 4. Similar to the evaluations for exercise maintenance, demographic variables at baseline which were significant in the univariate analyses (baseline BMI, income group, marital status, gender, smoking, hours spent watching television, family social support and friend social support) were entered into three separate models controlling for exercise level at baseline and treatment group to identify predictors exercise initiation at Years 2, 3 and 4. Four variables were significant predictors of exercise initiation at one or more time points. Exercise level at baseline was positively related to exercise initiation at Year 2 (OR = .23, CI = 1.71–2.90) and at Year 3 (OR = 1.71, CI = 1.23–2.38). Baseline income group (OR = 2.38, CI = 1.08–5.23), marital status (OR = 6.07, CI = 0.39–0.95) and smoking status (OR = 3.53, CI = 0.14–0.91) predicted exercise initiation at Year 4.

## Discussion

This study evaluated the demographic predictors of vigorous exercise initiation and maintenance in a community sample. To our knowledge, this is the first study to look at baseline demographic factors as predictors for exercise adoption and maintenance over a four-year period. This study found that demographic predictors were more consistent for exercise maintenance than exercise initiation over the four years evaluated in this study. Results showed that compared to those who did not maintain vigorous exercise, participants who maintained exercise over a 2, 3 and 4 year period were more likely to be employed, Caucasian, have a higher income, have more education, be a nonsmoker, watch less television and have a lower BMI. 

One of the interesting findings was that correlates of exercise initiation were less consistent than correlates of exercise maintenance over time. There did not appear to be a consistent pattern that described the associations between the predictors included in this study and vigorous exercise initiation. Although certain predictors were associated with exercise initiation at Years 2, 3 or 4, such as gender or BMI, none of the predictors were consistent over the years measured in the study. These results suggest that people in community populations who initiate vigorous exercise are a heterogeneous group, and there may be greater difficulties predicting who will initiate exercise. It could be much simpler to identify the participants who will be more likely to continue exercise, as compared to the participants who will adopt exercise. It is possible that changes in attitudes, life circumstances (such as sickness), new relationships, or variables that were not measured in this study may be better predictors of exercise adoption or maintenance. However, since we did not measure these variables, we can only speculate whether they may disrupt exercise patterns. The processes of vigorous exercise adoption may be better represented by theoretical understandings of exercise behavior, such as the health belief model [17], transtheoretical model [18,19], social cognitive theory [20], or the theory of planned behavior[21].

These results may have implications for designing and implementing exercise interventions. The results support others studies that suggest targeting exercise maintenance interventions at the lower income participants. However, to achieve the Healthy People 2010 objectives by increasing participation rate in vigorous exercise, interventions need to be designed to promote exercise adoption. Unfortunately, the present study's results only suggest that we can not characterize this group well. This lack of characterization of this group may actually be a benefit for interventions. Ethnic group, gender, and socio-economic status did not predict exercise initiation in this study. This suggests that each group was as likely to begin a vigorous exercise program. This is good news as exercise initiation may not be limited to those who can afford health clubs and trainers, or those who are the member of a specific ethnic group or gender. 

Although previous studies have stratified participants by gender [16], no interaction was found in this study between gender and exercise status. The only associations with gender were seen during Year 3, when fewer women than men maintained exercise and fewer women than men initiated exercise. It is possible that the gender by exercise interaction was not significant due to the limitation of exercise in the vigorous activity range, versus the moderate or mild activity range. One hypothesis is that the predictors of exercise adoption that are gender specific may have to do with moderate or minimal exercise adoption. 

Strengths and limitations of the present study should be recognized. This is one of a few studies that evaluate predictors of exercise initiation, and the first to evaluate exercise initiation over a four-year period. In addition, the sample is large, diverse, and includes longitudinal measurements. This study did not evaluate the predictors of moderate exercise initiation or maintenance. Thus, these results can not be directly interpreted for public health interventions targeted at increasing moderate activity in the general population. In addition, this study may also be affected by a selection bias. The participants in this study volunteered for a study on weight gain prevention, for which the participants may have expected to include a message on increasing exercise. Of note, 49% of the sample reported vigorous exercise more than 3 times per week at baseline, which is much higher than the 15–25% reported in national surveillance studies [6,2]. Although treatment status was controlled for in the analyses, these participants were recruited for an intervention trial, rather than a cohort trial, which could also contribute to a selection bias. In addition, the measures in this study are self-report and include few theoretically based variables. It is possible that the self-report nature of exercise in this study may have allowed over-reporting of vigorous exercise. 

Considering these limitations, this study does add to the knowledge base about who initiates and maintains vigorous exercise. The results suggest that vigorous exercise maintenance interventions should continue to target low-income populations and that interventions could incorporate messages regarding smoking, weight control, and television. We found that those who adopt vigorous exercise are a more heterogeneous group, and that no one group is more likely to adopt exercise than the others. This suggests that vigorous exercise intervention messages could be more broadly based. This study also suggests that further research is needed to identify participants and effective interventions for those who begin exercise programs. 

## Competing interests

None declared. 

## Authors contributions

KB conceptualized the study, planned and executed the analyses, interpreted the results, and drafted the manuscript. RJ participated in conceptualization of the study, interpretation of the results and assisted in drafting the manuscript. SF participated in conceptualization of the study, interpretation of the results and assisted in drafting the manuscript. All authors read and approved the final manuscript. 
